# Impact of SARS‐CoV‐2 infection on pain crisis and acute chest syndrome in patients with sickle cell anemia: A retrospective multi‐cohort study based on US national data from 2020 to 2022

**DOI:** 10.1002/jha2.840

**Published:** 2024-02-29

**Authors:** Juan Alvarado, Keval Yerigeri, Anita Boakye, Christina Randolph, Aparna Roy, Grace Onimoe

**Affiliations:** ^1^ MetroHealth Medical System Cleveland Ohio USA; ^2^ Case Western University Cleveland Ohio USA

**Keywords:** acute chest syndrome, COVID‐19, pain, SARS‐Cov‐2, sickle cell anaemia, sickle cell disease

## Abstract

COVID‐19 infection has been a significant contributor to global morbidity and mortality, especially among those patients with chronic diseases. The Centers for Disease Control and Prevention have classified sickle cell disease (SCD) as a condition that increases the risk of severe illness from COVID‐19 infection.

A retrospective study was conducted using the TRiNetX health research network database to identify SCA patients ( HbSS, Sbeta‐thalassemia zero) who had SARS‐CoV‐2 infection over 2 years; these were compared with similar patients who did not have the infection in terms of demographics, pain control, and laboratory parameters

COVID‐19 illness impacts [ain crises and ACS, and prior vaccination against influenza and COVID‐19 may represent a protective factor for developing pain crises.

## Graphical abstract

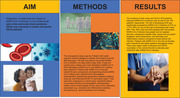

## 1 INTRODUCTION

COVID‐19 caused by the severe acute respiratory syndrome coronavirus 2 (SARS‐CoV‐2) pathogen manifests as an inflammatory response that can lead to multi‐organ damage/failure [1]. Declared a pandemic by the World Health Organization in March 2020, it has resulted in an upheaval of health sectors worldwide. The costs of the pandemic have surpassed 10 trillion dollars in foregone goods and services, reduced labor force participation, disruption to education, and restrictions on social functions and cultural exchange [2]. Over 40% of adults report that their families have lost jobs, work hours, or work‐related income due to the outbreak [3].

According to the Center for Systems Science and Engineering at Johns Hopkins University, there have been over 6 million deaths attributable to COVID‐19 in the United States as of 2022. The Centers for Disease Control claim that hospitalization and mortality is markedly higher in the 30–39 and 85+ age groups compared to those who are less than 30 years of age. Differences in immune responses between children and adults contribute to the variance in severity and hospitalization rates. Adults have suppressed adaptive immunity and hyperactivity of the innate immune response in severe infections, causing severe inflammation. Conversely, children have a strong immune response from frequent viral infections and live vaccines. They are more likely to gain early control of a SARS‐CoV‐2 infection and exhibit milder symptoms [4]. Adults with end‐organ disease (e.g., diabetes, chronic obstructive pulmonary disease, heart failure, and chronic kidney disease) are at increased risk for severe COVID‐19 and repeat admissions for complications [5]. This finding is mirrored in children with severe comorbidities such as obesity, diabetes, heart disease, and seizure disorders [6].

COVID‐19 infection has been a significant contributor to global morbidity and mortality, especially among those patients with chronic diseases. The Centers for Disease Control and Prevention have classified sickle cell disease (SCD) as one of the conditions considered to increase risk of severe illness from COVID‐19 infection. SCD by causing polymerization of hemoglobin, deformity of red blood cells, microvascular occlusion, and diffuse vascular endothelial damage places patients at high risk of thrombosis, end‐organ damage, and infections due to functional asplenia [7, 8]. This risk could be compounded by the uncontrolled release of pro‐inflammatory cytokines in COVID‐19 infection, as cytokine release mediates vascular injury and platelet activation [1].

Individuals with SCD over the age of 50 with pre‐existing cardiopulmonary or renal comorbidity are at high risk of death irrespective of genotype; additional risk factors include high serum creatinine, lactate dehydrogenase, and D‐dimer levels upon presentation. Reports of disease‐modifying drugs such as hydroxyurea, crizanlizumab, voxelotor, and l‐glutamine are higher in not hospitalized patients. Hydroxyurea therapy has been shown to lower mortality in patients with SCD and concomitant SARS‐CoV‐2 infection [9]. Hoogenboom et al. show that most patients with SCD and COVID‐19 have mild disease and a low risk of death, but patients with certain SCD‐related comorbidities, such as acute pain crisis, ACS, or both, are more likely to be hospitalized and require escalated care [10]. Identifying high‐risk individuals earlier in their disease course could expedite treatment and decrease morbidity/mortality, therefore, the overarching aim of this study is to identify whether the incidence, morbidity, and mortality of ACS and pain crises, two of the most common SCD‐related complications, increase in the presence of COVID‐19 infection using a large‐scale US database.

## 2 METHODS

This study is a retrospective analysis of cohorts drawn from the TriNetX USA health research network. TriNetX compiles electronic medical record data from over 100 healthcare organizations encompassing the United States, Europe, Brazil, and South Korea. Patients' data are de‐identified as per Health Insurance Portability and Accountability Act (HIPAA) and General Data Protection Regulation guidelines.

Six study cohorts (Table S1) were created on the TriNetX USA network by using the query builder function identifying ICD‐10 codes of the conditions/diagnostics of interest (SCD, pain crisis, ACS, SARS‐CoV‐2 infection). Inclusion and exclusion criteria were organized by using the operators AND OR MUST HAVE and CANNOT HAVE incorporated in the same platform.

The inclusion criteria are as follows:

- age 1–50 years old;
- sickle cell disease (SCD), genotype Hb‐SS or Sβ0‐thal;
- diagnosis of pain crisis or acute chest syndrome;
- available report of COVID‐19 status (positive/negative) at the same time of the pain crisis/ACS diagnosis, within May 2020 to May 2022.

The exclusion criteria are as follows:

- children with SCD and multisystem inflammatory syndrome (MIS‐C) as this condition presents weeks after the SARS‐CoV‐2 infection;
- patients with SCD genotype Hb‐SC and Sβ+ as these individuals tend to have milder clinical phenotypes, hence less incidence and severity of SCD complications (Baba et al., 2019).

The six created cohorts are pain crisis with/without SARS‐CoV‐2, ACS with/without SARS‐CoV‐2, and SARS‐CoV‐2 without pain crisis/ACS. The SARS‐CoV‐2 infection had to be reported at the same time the pain crisis/ACS was diagnosed.

All the cohorts were matched for demographic characteristics—age group (<18 years old, ≥18 years old), gender, race, and ethnicity—before comparing the groups and running statistical analysis; additionally, mutually exclusive diagnosis (ICD‐10 codes) were used to capture genuine cases of SCD, pain crisis, ACS, and SARS‐CoV‐2 infection. Patient data drawn from TriNetX included demographic characteristics, health insurance status, influenza and COVID‐19 vaccination status, level of service, baseline laboratory results, disease modifying drugs use, laboratory outcomes, pain control regimens, procedures (transfusion), and mortality.

Of note, Table S2 presents the description of the study cohorts as well as a study flowchart illustrating how the different cohorts were compared.

To investigate the reason why despite SARS‐CoV‐2 infection certain SCD patients do not develop pain crisis/ACS, patients with SARS‐CoV‐2 and pain crisis/ACS were compared to their counterparts (SARS‐CoV‐2 without pain crisis/ACS). Demographic characteristics (Table 1), baseline laboratory parameters, disease‐modifying drug use, and influenza/COVID‐19 vaccination status were obtained. All these parameters had to be reported at least 6 months before the diagnosis of (1) pain crisis/ACS with concomitant SARS‐CoV‐2 or (2) SARS‐CoV‐2 infection alone (Table 4). Laboratory parameters include hemoglobin and reticulocyte percentage. Disease‐modifying drugs include hydroxyurea, voxelotor, l‐glutamine, and crizanlizumab. Mean and standard deviation were calculated for age and laboratory values; *p*‐values were determined via two‐sample *t*‐tests. Total patient count and proportion were calculated for the other variables.

**TABLE 1:** Demographic information of patients with and without pain crises/acute chest syndrome (ACS) and concomitant severe acute respiratory syndrome coronavirus 2 (SARS‐CoV‐2) infection.

		SARS‐CoV‐2			SARS‐CoV‐2		
		Pain crisis	Noncrisis	*p*‐value	ACS	Non‐ACS	*p*‐value
Age	Mean age, years ± SD	29.2 ± 11.4	27.5 ± 13.5	<0.0001	24.5 ± 11.8	26.8 ± 13.2	<0.0001
	<18 years, *n* (%)	201 (16.17)	1563 (25.98)	<0.0001	552 (29.98)	2502 (26.87)	0.006
	≥19 years, *n* (%)	1027 (82.62)	4353 (72.35)	<0.0001	1240 (67.36)	6632 (71.22)	0.0009
Gender	Female, *n* (%)	678 (54.54)	4596 (76.38)	<0.0001	915 (49.70)	6437 (68.79)	<0.0001
	Male, *n* (%)	564 (45.37)	1412 (23.47)	<0.0001	918 (49.86)	2902 (31.01)	<0.0001
Ethnic group	Not Hispanic or Latino, *n* (%)	1044 (83.99)	5042 (83.79)	0.87	1608 (87.34)	7796 (88.32)	<0.0001
	Hispanic or Latino, *n* (%)	31 (2.49)	625 (10.39)	<0.0001	43 (2.34)	722 (7.72)	<0.0001
	Unknown ethnicity, *n* (%)	168 (13.52)	350 (5.82)	<0.0001	190 (10.32)	839 (8.97)	0.06
Race	Black or African American, *n* (%)	1089 (87.62)	2593 (43.09)	<0.0001	1689 (91.74)	5599 (59.84)	<0.0001
	White, *n* (%)	78 (6.28)	2630 (43.71)	<0.0001	31 (1.68)	2718 (29.05)	<0.0001
	Asian, *n* (%)	10 (0.81)	106 (1.76)	0.01	–	113 (1.21)	<0.0001
	American Indian, *n* (%)	–	10 (0.17)	0.15	10 (0.54)	13 (0.14)	0.0005
	Native Hawaiian, *n* (%)	–	10 (0.17)	0.15	10 (0.54)	10 (0.11)	<0.0001
	Unknown race, *n* (%)	70 (5.63)	680 (11.30)	<0.0001	117 (6.36)	912 (9.75)	<0.0001
Lack of health insurance, *n* (%)		10 (0.81)	–	<0.0001	–	–	–

To investigate the differences in morbidity and mortality of pain crisis/ACS with and without SARS‐CoV‐2 infection, patients with pain crisis/ACS and SARS‐CoV‐2 were then compared to their counterparts (pain crisis/ACS without concomitant infection). Demographic characteristics (Table 3), laboratory outcomes, and clinical outcomes were obtained. These parameters had to be reported up to 1‐month after the diagnosis of (1) pain crisis/ACS with concomitant infection or (2) pain crisis/ACS alone (Tables 5 ). Laboratory outcomes include hemoglobin, reticulocyte %, leukocyte count, lactate dehydrogenase (LDH), C‐reactive protein (CRP), erythrocyte sedimentation rate (ESR), and ferritin. Clinical outcomes include use of pain regimens (opioids, nonopioid analgesia, nonsteroidal anti‐inflammatory drugs [NSAIDs], and opioid antagonists), blood transfusion, mortality, and settings of care—emergency department, inpatient admission, intensive care unit (ICU), and subsequent hospital care (re‐admission). Critical lab parameters were determined based on clinical applications; patient counts with critical lab values were enumerated for each cohort. Relative risk with *p*‐value was calculated comparing the groups with and without SARS‐CoV‐2 infection. Though the period of the study did include the time frame in which Omicron was identified, the database does not include the subtype of COVID‐19 that the patients had, so this information could not be obtained.

The primary endpoint of the study is the effect of SARS‐CoV‐2 infection on the incidence of pain crises and ACS. Secondary endpoints include acuity of care setting, need for disease‐modifying therapy, the effect of COVID‐19 and influenza vaccination status, transfusion burden, critical lab values, length of hospital stay, and mortality.

## 3 RESULTS

After matching, a total of 16,341 patients with sickle cell anemia were identified who met the inclusion criteria for this study. The majority of subjects were adults (19–50 years old), the most common race was African American, and the most common ethnic group was Not Hispanic or Latino. Only 20 patients were identified as not having medical insurance.

Table 1 shows the demographic characteristics of the SCD patients with concomitant SARS‐CoV‐2 infection and pain crisis or ACS and those without pain crisis or ACS.

Table 2 shows the demographic characteristics of the SCD patients who developed pain crisis or ACS with concomitant SARS‐CoV‐2 infection and those without the infection.

**TABLE 2:** Demographic information of patients with and without severe acute respiratory syndrome coronavirus 2 (SARS‐CoV‐2) infection who developed pain crises/acute chest syndrome (ACS).

		Pain crisis			ACS		
		SARS‐CoV‐2	Non‐SARS‐CoV‐2	*p*‐value	SARS‐CoV‐2	Non‐SARS‐CoV‐2	*p*‐value
Age	Mean age, years ± SD	29.2 ± 11.5	28.5 ± 11.7	0.14	24.7 ± 11.7	24.9 ± 11.4	0.71
	<18 years, *n* (%)	198 (16.02)	296 (18.79)	0.06	551 (28.86)	180 (28.44)	0.84
	≥19 years, *n* (%)	1022 (82.69)	1254 (79.62)	0.04	1309 (68.57)	443 (69.98)	0.51
Gender	Female, *n* (%)	673 (54.45)	798 (50.68)	0.05	955 (50.03)	305 (48.18)	0.42
	Male, *n* (%)	563 (44.55)	776 (49.27)	0.05	950 (49.76)	328 (51.82)	0.37
Ethnic group	Not Hispanic or Latino, *n* (%)	1040 (84.14)	1161 (73.71)	<0.0001	1678 (87.89)	538 (84.99)	0.06
	Hispanic or Latino, *n* (%)	29 (2.35)	43 (2.73)	0.52	44 (2.31)	30 (4.74)	0.002
	Unknown ethnicity, *n* (%)	167 (13.51)	371 (23.56)	<0.0001	187 (9.79)	65 (10.27)	0.73
Race	Black or African American, *n* (%)	1083 (87.62)	1,333 (84.64)	0.02	1758 (92.09)	570 (90.05)	0.11
	White, *n* (%)	77 (6.23)	63 (4)	0.007	31 (1.62)	17 (2.69)	0.09
	Asian, *n* (%)	10 (0.81)	10 (0.64)	0.52	–	–	–
	Unknown race, *n* (%)	70 (5.66)	169 (10.73)	<0.0001	116 (6.08)	46 (7.27)	0.29
	Native Hawaiian, *n* (%)	–	–	–	10 (0.52)	–	0.07
Lack of health insurance, *n* (%)		10 (0.72)	–	0.0006	–	10 (1.57)	<0.0001

Patients with SCD and concomitant SARS‐CoV‐2 infection were compared with patients without the infection to determine the outcomes of pain crisis and ACS. The incidence of pain crises with accompanying infection was 34 per 100 patients, while the incidence of ACS with concomitant infection was 27 per 100 patients. SARS‐CoV‐2 significantly increased the risk of developing ACS (2.57% vs. 1.17% [*p* < 0.0001], relative risk [RR]: 2.2 [95% confidence interval [CI]: 1.72–2.82]), but not the risk for developing pain crises (4.35% vs. 4.18% [*p* = 0.71], RR: 1.04 [95% CI: 0.85–1.27]).

The cohort with SARS‐CoV‐2 and subsequent pain crisis had higher use of disease‐modifying agents (hydroxyurea, voxelotor, l‐glutamine, and crizanlizumab) in the 6 months prior to infection than the cohort without pain crisis (*p* < 0.0001). The former cohort also had lower baseline hemoglobin levels (9.57 ± 2.15 vs. 11.5 ± 2.31, *p* < 0.0001) and significantly lower influenza and COVID‐19 immunization rates (*p* < 0.0001) (Table 3) compared to the group who did not develop the pain crisis.

**TABLE 3:** Baseline information of patients with and without pain crisis and concomitant severe acute respiratory syndrome coronavirus 2 (SARS‐CoV‐2) infection.

		SARS‐CoV‐2			SARS‐CoV‐2		
		Pain crisis	Noncrisis	*p*‐value	ACS	Non‐ACS	*p*‐value
Laboratory features	Hemoglobin, g/dL, mean ± SD^a^	9.57 ± 2.15	11.5 ± 2.31	<0.0001	8.86 ± 1.86	10.7 ± 2.41	<0.0001
	Reticulocyte, %, mean ± SD^a^	7.04 ± 6.53	7.22 ± 6.37	0.75	10.8 ± 7.53	8.26 ± 6.44	<0.0001
Disease‐modifying therapy	Hydroxyurea, *n* (%)^a^	548 (44)	737 (12)	<0.0001	1535 (80)	3172 (33)	<0.0001
	Voxelotor, *n* (%)^a^	33 (3)	25 (0.7)	<0.0001	187 (10)	242 (3)	<0.0001
	L‐glutamine, *n* (%)^a^	37 (3)	49 (1)	<0.0001	219 (11)	354 (4)	<0.0001
	Crizanlizumab, *n* (%)^a^	10 (1)	10 (0.5)	<0.0001	154 (8)	204 (2)	<0.0001
Immunization status	Influenza vaccine, *n* (%)^a^	37 (3)	760 (12)	<0.0001	262 (14)	1248 (13)	0.35
	SARS‐CoV‐2 vaccine, *n* (%)^a^	68 (6)	881 (14)	<0.0001	310 (16)	1299 (13)	0.001

^a^ Reported up to 6 months prior to the diagnosis of pain crisis/ACS with concomitant SARS‐CoV‐2 or the diagnosis of the infection alone w/o pain crisis or ACS.

Up to 6 months previous to the infection, the SARS‐CoV‐2 cohort who developed ACS also had higher use of disease‐modifying agents (*p* < 0.0001) and lower hemoglobin levels (8.86 ± 1.86 vs. 10.7 ± 2.41, *p* < 0.0001) than its sister cohort without ACS. Of note, the reticulocyte percentage was higher in the ACS cohort (10.8 ± 7.53 vs. 8.26 ± 6.44, *p* < 0.0001). Interestingly, COVID‐19 vaccination rates were slightly higher in the group that developed ACS (16% vs. 13%, *p* < 0.0001). The report of influenza vaccination was similar in both groups (14% vs. 13%, *p* = 0.35) (Table 3).

The cohorts with pain crisis/ACS and concomitant SARS‐CoV‐2 infection were then compared to their counterparts without infection. Laboratory results up to 1 month after the diagnosis of pain crisis/ACS are listed in Table 4. Clinical outcomes are compared in Table 5 and detailed below.

**TABLE 4:** Laboratory outcomes of patients with pain crisis/acute chest syndrome (ACS) with and without concomitant severe acute respiratory syndrome coronavirus 2 (SARS‐CoV‐2) infection.

	Pain crisis			ACS		
	SARS‐CoV‐2	Non‐SARS‐CoV‐2	*p*‐value	SARS‐CoV‐2	Non‐SARS‐CoV‐2	*p*‐value
Hemoglobin, g/dL, mean ± SD^a^	9.01 ± 1.91	9.41 ± 2.16	0.004	8.7 ± 1.78	8.74 ± 1.74	0.80
Reticulocyte, %, mean ± SD^a^	6.57 ± 5.71	4.87 ± 4.46	0.22	9.96 ± 8.05	7.77 ± 4.10	0.04
Leukocytes, 10^3^/μL, mean ± SD^a^	12.59 ± 48.74	10.61 ± 5.04	0.49	11.43 ± 4.55	10.72 ± 5.59	0.08
LDH, U/L, mean ± SD^a^	586.52 ± 871.26	477.99 ± 472.06	0.3	513.87 ± 418.43	653.04 ± 634.21	0.03
CRP, mg/L, mean ± SD^a^	62.10 ± 85.63	70.66 ± 120.69	0.73	48.62 ± 68.79	45.00 ± 70.92	0.85
ESR, mm/h, mean ± SD^a^	43.14 ± 34.43	38.94 ± 33.47	0.67	41.82 ± 34.79	40.25 ± 31.06	0.91
Ferritin, ng/mL, mean ± SD^a^	1661.14 ± 2778.74	3595.41 ± 1181.19	0.06	1906.78 ± 2246.23	2106.76 ± 3049.73	0.60

Abbreviations: CRP, C‐reactive protein; ESR, erythrocyte sedimentation rate; LDH, lactate dehydrogenase.

^a^ Reported up to 1 month after the diagnosis of pain crisis/ACS with concomitant infection or the diagnosis of pain crisis/ACS alone w/o SARS‐CoV‐2 infection.

**TABLE 5:** Clinical outcomes of patients with pain crisis/acute chest syndrome (ACS) with and without concomitant severe acute respiratory syndrome coronavirus 2 (SARS‐CoV‐2) infection.

		Pain crisis				ACS			
		SARS‐CoV‐2	Non‐SARS‐CoV‐2	*p*‐value	RR (95% CI)	SARS‐CoV‐2	Non‐SARS‐CoV‐2	*p*‐value	RR (95% CI)
Setting of care	Emergency department, *n* (Risk %)^a^	118 (9.88)	115 (9.63)	0.84	1.03 (0.80–1.31)	134 (21.58)	73 (11.76)	<0.0001	1.84 (1.41–2.39)
	Hospital inpatient, *n* (Risk %)^a^	365 (30.57)	153 (12.81)	<0.0001	2.39 (2.01–2.83)	328 (52.82)	176 (28.34)	<0.0001	1.86 (1.61–2.16)
	Intensive care unit, *n* (Risk %)^a^	16 (1.34)	11 (0.92)	0.33	1.46 (0.68–3.12)	40 (6.44)	24 (3.87)	0.04	1.67 (1.02–2.73)
	Subsequent hospital care, *n* (Risk %)^a^	268 (22.45)	100 (8.38)	<0.0001	2.68 (2.16–3.32)	276 (44.44)	150 (24.16)	<0.0001	1.84 (1.56–2.17)
Pain control	Opioid analgesics, *n* (Risk %)^a^	728 (60.97)	423 (35.42)	<0.0001	1.72 (1.57–1.88)	461 (74.24)	296 (47.67)	<0.0001	1.56 (1.42–1.71)
	Nonopioid analgesics, *n* (Risk %)^a^	499 (41.79)	265 (22.19)	<0.0001	1.88 (1.66–2.13)	403 (64.89)	237 (38.16)	<0.0001	1.70 (1.52–1.91)
	NSAIDs, *n* (Risk %)^a^	184 (15.41)	98 (8.21)	<0.0001	1.88 (1.49–2.37)	219 (35.27)	111 (17.87)	<0.0001	1.97 (1.62–2.41)
	Opioid antagonists, *n* (Risk %)^a^	175 (14.66)	86 (7.20)	<0.0001	2.04 (1.59–2.60)	141 (22.71)	56 (9.02)	<0.0001	2.52 (1.89–3.36)
Critical laboratory outcomes	Hemoglobin ≤9 g/dL, *n* (Risk %)^a^	485 (40.62)	161 (13.48)	<0.0001	3.01 (2.57–3.53)	379 (61.03)	161 (25.93)	<0.0001	2.35 (2.03–2.72)
	Reticulocyte ≥3%, *n* (Risk %)^a^	46 (3.85)	12 (1.01)	<0.0001	3.83 (2.04–7.12)	75 (12.08)	70 (11.27)	0.66	1.07 (0.79–1.46)
	Leukocytes ≥10,000/μL, *n* (Risk %)^a^	480 (40.20)	185 (15.49)	<0.0001	2.59 (2.23–3.01)	360 (57.97)	178 (28.66)	<0.0001	2.02 (1.76–2.33)
	LDH ≥280 U/L, *n* (Risk %)^a^	181 (15.16)	49 (4.10)	<0.0001	3.69 (2.72–5.01)	158 (25.44)	73 (11.76)	<0.0001	2.16 (1.68–2.79)
	CRP ≥5 mg/L, *n* (Risk %)^a^	92 (7.71)	15 (1.26)	<0.0001	6.13 (3.58–10.52)	77 (12.39)	10 (1.61)	<0.0001	7.70 (4.02–14.74)
	ESR ≥30 mm/h, *n* (Risk %)^a^	28 (2.35)	10 (0.84)	0.003	2.80 (1.37–5.74)	20 (3.22)	10 (1.61)	0.06	2.00 (0.94–4.24)
	Ferritin ≥350 ng/mL, *n* (Risk %)^a^	80 (6.7)	26 (2.18)	<0.0001	3.08 (1.99–4.75)	101 (16.26)	54 (8.69)	<0.0001	1.87 (1.37–2.55)
Blood transfusion, *n* (Risk %)^a^		122 (10.22)	51 (4.27)	<0.0001	2.39 (1.74–3.28)	121 (19.49)	90 (14.49)	0.02	1.34 (1.05–1.73)
Death, *n* (Risk %)^a^		10 (0.84)	10 (0.84)	1.00	1.00 (0.42–2.39)	10 (1.61)	10 (1.61)	1.00	1.00 (0.42–2.39)

Abbreviations: CI, confidence interval; CRP, C‐reactive protein; ESR, erythrocyte sedimentation rate; LDH, lactate dehydrogenase; NSAIDs, nonsteroidal anti‐inflammatory drugs; RR, relative risk.

^a^ Reported up to 1 month after the diagnosis of pain crisis/ACS with concomitant infection or the diagnosis of pain crisis/ACS alone w/o SARS‐CoV‐2 infection.

Differences between the pain crisis cohort with concomitant SARS‐CoV‐2 infection versus those without infection are outlined as follows:

1. *Setting of care*

The cohort with concomitant SARS‐CoV‐2 infection had the same risk of requiring ER visits (9.88% vs. 9.63% [*p* = 0.84], RR: 1.03 [95% CI: 0.80–1.31]) and ICU services (1.34% vs. 0.92% [*p* = 0.33], RR: 1.46 [95% CI: 0.68–3.12]), but had more risk of requiring hospital inpatient services (30.57% vs. 12.81% [*p* < 0.0001], RR: 2.39 [95% CI: 2.01–2.83]) and subsequent hospital services (22.45% vs. 8.38% [*p* < 0.0001], RR: 2.68 [95% CI: 2.16–3.32]).

2. *Pain control*

The cohort with concomitant SARS‐CoV‐2 infection had more risk of requiring opioid analgesics (60.97% vs. 35.42% [*p* < 0.0001], RR: 1.72 [95% CI: 1.57–1.88]), nonopioid analgesics (41.79% vs. 22.19% [*p* < 0.0001], RR: 1.88 [95% CI: 1.66–2.13]), NSAIDs (15.41% vs. 8.21% [*p* < 0.0001], RR: 1.88 [95% CI: 1.49–2.37]), and opioid antagonists (14.66% vs. 7.20% [*p* < 0.0001], RR: 2.04 [95% CI: 1.59–2.60]).

3. *Critical laboratory outcomes*

The cohort with concomitant SARS‐CoV‐2 infection had more risk of a hemoglobin level ≤ 9 g/dL (40.62% vs. 13.48% [*p* < 0.0001], RR: 3.01 [95% CI: 2.57‐3.53]), reticulocyte level ≥ 3% (3.85% vs. 1.01% (*p* < 0.0001], RR: 3.83 [95% CI: 2.04–7.12]), leukocyte count ≥ 10,000/μL (40.20% vs. 15.49% [*p* < 0.0001], RR: 2.59 [95% CI: 2.23–3.01]), LDH level ≥ 280 U/L (15.16% vs. 4.10% [*p* < 0.0001], RR: 3.69 [95% CI: 2.72–5.01]), CRP level ≥ 5 mg/L (7.71% vs. 1.26% [*p* < 0.0001], RR: 6.13 [95% CI: 3.58–10.52]), ESR level ≥ 30 mm/h (2.35% vs. 0.84% [*p* = 0.003], RR: 2.80 [95% CI: 1.37–5.74]), and ferritin level ≥ 350 ng/mL (6.7% vs. 2.18% [*p* < 0.0001], RR: 3.08 [95% CI: 1.99–4.75]).

4. *Blood transfusion*

The cohort with concomitant SARS‐CoV‐2 infection had more risk of requiring blood transfusion (10.22% vs. 4.27% [*p* < 0.0001], RR: 2.39 [95% CI: 1.74–3.28]).

5. *Death*

The cohort with concomitant SARS‐CoV‐2 infection had similar mortality (0.84% vs. 0.84% [*p* = 1], RR: 1 [95% CI: 0.42–2.39]).

Differences between the ACS cohort with concomitant SARS‐CoV‐2 infection versus those without infection are outlined as follows:

6. *Setting of care*

The cohort with concomitant SARS‐CoV‐2 infection had more risk of requiring ED visits (21.58% vs. 11.76% [*p* < 0.0001], RR: 1.84 [95% CI: 1.41–2.39]), hospital inpatient services (52.82% vs. 28.34% [*p* < 0.0001], RR: 1.86 [95% CI: 1.61–2.16]), ICU services (6.44% vs. 3.87% [*p* = 0.04], RR: 1.67 [95% CI: 1.02–2.73]), and subsequent hospital services (44.44% vs. 24.16% [*p* < 0.0001], RR: 1.84 [95% CI: 1.56–2.17]).

7. *Pain control*

The cohort with concomitant SARS‐CoV‐2 infection had more risk of requiring opioid analgesics (74.24% vs. 47.67% [*p* < 0.0001], RR: 1.56 [95% CI: 1.42–1.71]), nonopioid analgesics (64.89% vs. 38.16% [*p* < 0.0001], RR: 1.70 [95% CI: 1.52–1.91]), NSAIDs (35.27% vs. 17.87% [*p* < 0.0001], RR: 1.97 [95% CI: 1.62–2.41]), and opioid antagonists (22.71% vs. 9.02% [*p* < 0.0001], RR: 2.52 [95% CI: 1.89–3.36]).

8. *Critical laboratory outcomes*

The cohort with concomitant SARS‐CoV‐2 infection had more risk of having a hemoglobin level ≤ 9 g/dL (61.03% vs. 25.93% [*p* < 0.0001], RR: 2.35 [95% CI: 2.03–2.72]), leukocyte count ≥ 10,000/μL (57.97% vs. 28.66% [*p* < 0.0001], RR: 2.02 [95% CI: 1.76‐2.33]), LDH level ≥ 280 U/L (25.44% vs. 11.76% [*p* < 0.0001], RR: 2.16 [95% CI: 1.68–2.79]), CRP level ≥ 5 mg/L (12.39% vs. 1.61% [*p* < 0.0001], RR: 7.70 [95% CI: 4.02–14.74]), and ferritin level ≥ 350 ng/mL (16.26% vs. 8.69% [*p* < 0.0001], RR: 1.87 [95% CI: 1.37–2.55]), but had the same risk of having a reticulocyte level ≥ 3% (12.08% vs. 11.27% [*p* 0.66], RR: 1.07 [95% CI: 0.79–1.46]), and ESR level ≥30 mm/h (3.22% vs. 1.61% [*p* = 0.06], RR: 2 [95% CI: 0.94–4.24]).

9. *Blood transfusion*

The cohort with concomitant SARS‐CoV‐2 infection had the same risk of requiring blood transfusion (19.49% vs. 14.49% [*p* = 0.02], RR: 1.34 [95% CI: 1.05–1.73]).

10. *Death*

The cohort with concomitant SARS‐CoV‐2 infection had the same risk of death (1.61% vs. 1.61% [*p* = 1], RR: 1 [95% CI: 0.42–2.390]).

## 4 DISCUSSION

Due to the limited large‐scale data on sickle cell outcomes and complications amidst the COVID‐19 pandemic, this study evaluates the impact of SARS‐CoV‐2 on two SCD‐related complications: pain crises and ACS using a large US‐based data set. Patients with genotypes Hb‐SS or Sβ0‐thal, which are considered the more severe forms of SCD, are emphasized [11]. The findings of the present study show that SARS‐CoV‐2 did not increase the risk of pain crisis but did increase the risk of ACS. The former is not consistent with a study by Mucalo et al., which suggests that COVID‐19 infection may be a trigger for pain crisis in SCD patients [12].

Mucalo et al. analyzed the US‐based SECURE‐SCD registry and identified 750 cases of COVID‐19 illness in patients with SCD. Pain crisis and ACS were the most common presenting symptoms (67% and 29% in adults, respectively) followed by nephropathy, overt stroke, and pulmonary hypertension. Note that 40% of children and 60% of adults required inpatient admission, and 6% of children and 9% of adults required ICU level of care. The mortality rate for the total cohort was 2.5%, higher than the present study which had a mortality rate of 0.84% in the pain crisis cohort and 1.61% in the ACS cohort [12]. This contrasts significantly with the mortality rate of 10.6% reported in a small prospective study by Minniti et al. [9].

In another study of the SECURE‐SCD registry, Panepinto et al. identified 178 COVID‐19 patients with SCD; 6% of patients were asymptomatic, 54% had mild disease, and 40% had moderate to critical disease severity. Sixty‐nine percent required inpatient admission and 11% needed ICU level of care [13]. Similarly, the prospective study by Minniti et al. reported that 76% of patients required hospital admission [9]. These rates were higher than the findings of the present TriNetX‐based cohorts, with only 31% of the concomitant SARS‐CoV‐2/pain crisis and 44% of the SARS‐CoV‐2/ACS patients requiring inpatient services. A cohort from Panepinto et al. also showed a higher mortality rate of 7%; pulmonary hypertension, stroke, and diminished renal function were common comorbidities in deceased patients. Thirty‐two percent of patients developed ACS, but a focused analysis of this cohort is unavailable [13].

Hoogenboom et al. performed a comprehensive review of the literature studying SCD in COVID‐19 patients and identified 2290 patients across 71 studies (including Mucalo et al. and Panepinto et al.) from January 2020 to October 2021. From the aggregate data, 62% of patients presented with pain crisis and 47% with ACS. Most studies had hospitalization rates greater than 40% and as high as 72% across the case studies [10]. This contrasts in particular with our SARS‐CoV‐2/pain crisis cohort. The difference may be attributed to selection bias in a retrospective study and the unique biases associated with the TriNetX system.

This study does exhibit an increased incidence of ACS and related interventions in SCD patients with SARS‐CoV‐2. Several case studies have previously suggested associations between COVID‐19 and ACS. ACS remains a leading cause of ICU admissions and mortality in SCD patients and is a complex pulmonary condition often triggered by an atypical bacteria or less commonly by a virus [14]. Han et al. confirm an increase in ACS but not pain crisis in patients with COVID‐19 [15]. Several factors may contribute to this outcome. Hospitalization due to other SCD complications such as fever, superimposed infections, avascular necrosis, and stroke are not captured in this study. Protective measures against pain crises (e.g., utilization of SCD‐modifying agents) may have reduced pain crisis incidence during the pandemic. Social isolation precautions such as masking, stay‐at‐home orders, and virtual education limited the spread of infection. Sayad et al. also hypothesize that hydroxyurea leads to favorable pain outcomes [8].

Disease‐modifying agents are known to have multiple benefits in SCD. In a prospective study of 66 individuals with SCD and concomitant SARS‐CoV‐2 by Minniti et al., the use of disease‐modifying agents was reported in 69% of not hospitalized and in 49% of hospitalized patients; mortality was not reported [9]. The use of disease‐modifying agents was also suboptimal in the present study, reported in 80% of ACS patients and only in 44% of pain crisis patients; however, TriNetX is unable to capture adherence to medications, duration of treatment, or combination therapies.

During the pandemic, vaccination rates were reported to be lower in minority populations in which SCD majorly occurs [16]. A study of adult patients with SCD in an urban population in Illinois found that COVID‐19 vaccination rates were lower among patients with SCD than among the general African‐American population [15]. With regard to the pediatric population, in a study by Eyssette‐Guerreau et al., during the Omicron wave in France, only two of 17 patients that were admitted to the hospital with severe COVID‐19 ± SDC complications had at least one dose of the vaccine [17].

There is controversy regarding the benefit of vaccination in SCD patients. In a study by Prince et al., COVID‐19 immunization reduced the risk of hospitalization in 5‐ to 11‐year‐old patients during the Omicron wave, and the risk of hospitalization and severe illness in 12‐ to 18‐year‐old patients during the Delta and Omicron waves [18]. In the same study by Eyssette‐Guerreau et al., patients with severe COVID‐19 and SCD during the Omicron wave required noninvasive respiratory support, transfusions, and longer hospital stays, suggesting that vaccination could be potentially beneficial [17]. These two studies contrast with the findings of Derdevet et al. on 88 adult SCD patients that showed that SARS‐CoV‐2 infection (during the Omicron wave) does not cause more severe disease in patients with weak or nonprotection against COVID‐19 [19].

In our study, COVID‐19 vaccination rates were low (less than 20%) in every cohort, yet it demonstrated that previous immunization may have played an important role in preventing pain crises, finding also true for the influenza vaccine [20]. Due to the timespan of the study, our data likely include patients who did not have access to COVID‐19 vaccines that could potentially underestimate the true benefit of immunizations. Further research regarding the benefits of vaccination in pediatric and adult patients with SCD is a priority to be explored as strategies to increase vaccination rates could be implemented.

Our study has several potential limitations. First, in a retrospective study, data entry and collection methods are not established before study operations [21]. This is further complicated in assessments of the TriNetX database because data quality cannot be retroactively ensured by investigators. TriNetX provides access to a large data set, but it poses unique limitations. It does not capture individual socioeconomic factors that influence health outcomes. It also collects data when patients present to participating healthcare organizations (HCOs); admissions to other institutions may account for the lower admission rates of this study. Second, participating HCOs are mainly large academic centers, skewing the study population. Third, loss to follow‐up is also unaccounted for in data analysis and exclusion criteria [22]. These confounding variables are not inconsequential and must be factored in when considering the generalizability of this study's results. Fourth, this study looked exclusively at pain crises and ACS with genotypes Hb‐SS or Sβ0‐thal, thus ignoring other genotypes in association with COVID‐19. Additionally, although the period of the present study does include the Delta and Omicron variants of SARS‐CoV‐2, our database does not specify which variant each patient had.

This study contributes to the field by showing the impact of COVID‐19 on SCD‐specific outcomes, which is important to understand for patient care, especially as COVID‐19 variants continue to unfold. The study also contributes to research and management strategies for sickle cell disease as it reports on the effect of a disease of pandemic propensity (COVID‐19) on SCD, hence providing crucial information for practitioners on how to anticipate and plan response in treating these patients. Future directions include patient‐by‐patient chart review to better understand medical and psychosocial factors influencing health outcomes, exploration of the mental health effects of the pandemic on patients with concomitant SCD and COVID‐19, and development of actionable policies and advocacy for COVID‐19 vaccination.

## AUTHOR CONTRIBUTIONS

Grace Onimoe conceived the study, contributed to the analysis and interpretation of the data, and wrote the manuscript. Juan Alvarado conceived the study; contributed to the acquisition, analysis, and interpretation of data; and wrote the manuscript. Keval Yerigeri, Anita Boakye, Christina Randolph, and Aparna Roy contributed to the interpretation of data and wrote the manuscript. All authors had full access to all the data in the study.

## CONFLICT OF INTEREST STATEMENT

The authors declare no conflicts of interest.

## FUNDING INFORMATION

Department of Pediatrics, MetroHealth System; The John Patrick Carey Foundation

## ETHICS STATEMENT AND PATIENT CONSENT

All the data from this study were obtained from the TriNetX USA network from de‐identified patients compliant with HIPAA regulations. No personal information was obtained. Access to specific HCOs was not requested. No IRB was required. No patient consent was required.

## Supporting information

Supporting Information

## Data Availability

The data that support the findings of this study are available on request from the corresponding author upon reasonable request.
