# Supplementary material for: Impact of SARS‐CoV‐2 infection on pain crisis and acute chest syndrome in patients with sickle cell anemia: A retrospective multi‐cohort study based on US national data from 2020 to 2022
Source: EJHaem. 2024 Feb 29;5(2):299–307. doi: 10.1002/jha2.840 (PMC11020112; doi:10.1002/jha2.840)
Supplement: Supplementary file 1 — Supporting Information [file JHA2-5-299-s001.docx]

| **Supplementary Table 1. Study cohorts and inclusion criteria** | | | | |
| --- | --- | --- | --- | --- |
| **Cohorts** | | **Number of Patients** | | **HCOs** |
|  |  | **Before matching *** | **After matching *** |  |
| **1** | SCD patients with pain crisis with SARS-CoV-2 infection | 1,236 | 1,194 | 45 |
| **2** | SCD patients with pain crisis without SARS-CoV-2 infection | 1,575 | 1,194 | 51 |
| **3** | SCD patients with ACS with SARS-CoV-2 infection | 1,909 | 621 | 43 |
| **4** | SCD patients with ACS without SARS-CoV-2 infection | 633 | 621 | 46 |
| **5** | SCD patients with SARS-CoV-2 infection without pain crisis | 6,153 | 4,211 | 50 |
| **6** | SCD patients with SARS-CoV-2 infection without ACS | 9,649 | 7,700 | 50 |
| ACS: acute chest syndrome; HCOs: healthcare organizations contributing data to each cohort; SCD: sickle cell disease  *: all cohorts were matched for age group (0-18 years, ≥18 years), gender, race and ethnicity | | | | |

| **Supplementary Table 2. Study flowchart** | |
| --- | --- |
| **Comparisons between cohorts** | **Objectives** |
| 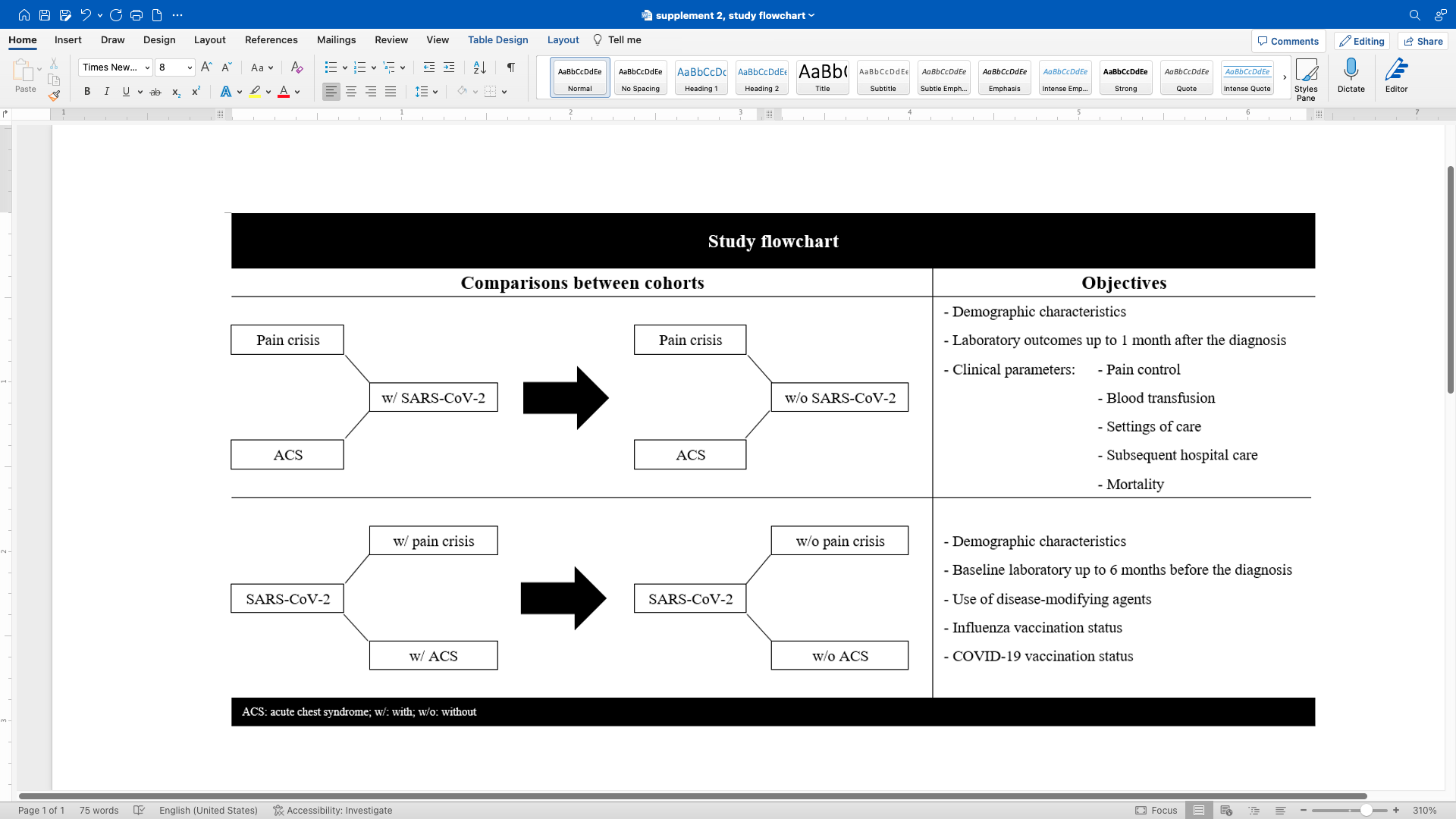 | - Demographic characteristics  - Laboratory outcomes up to 1 month after the diagnosis  - Clinical parameters: - Pain control  - Blood transfusion  - Settings of care  - Subsequent hospital care  - Mortality |
| 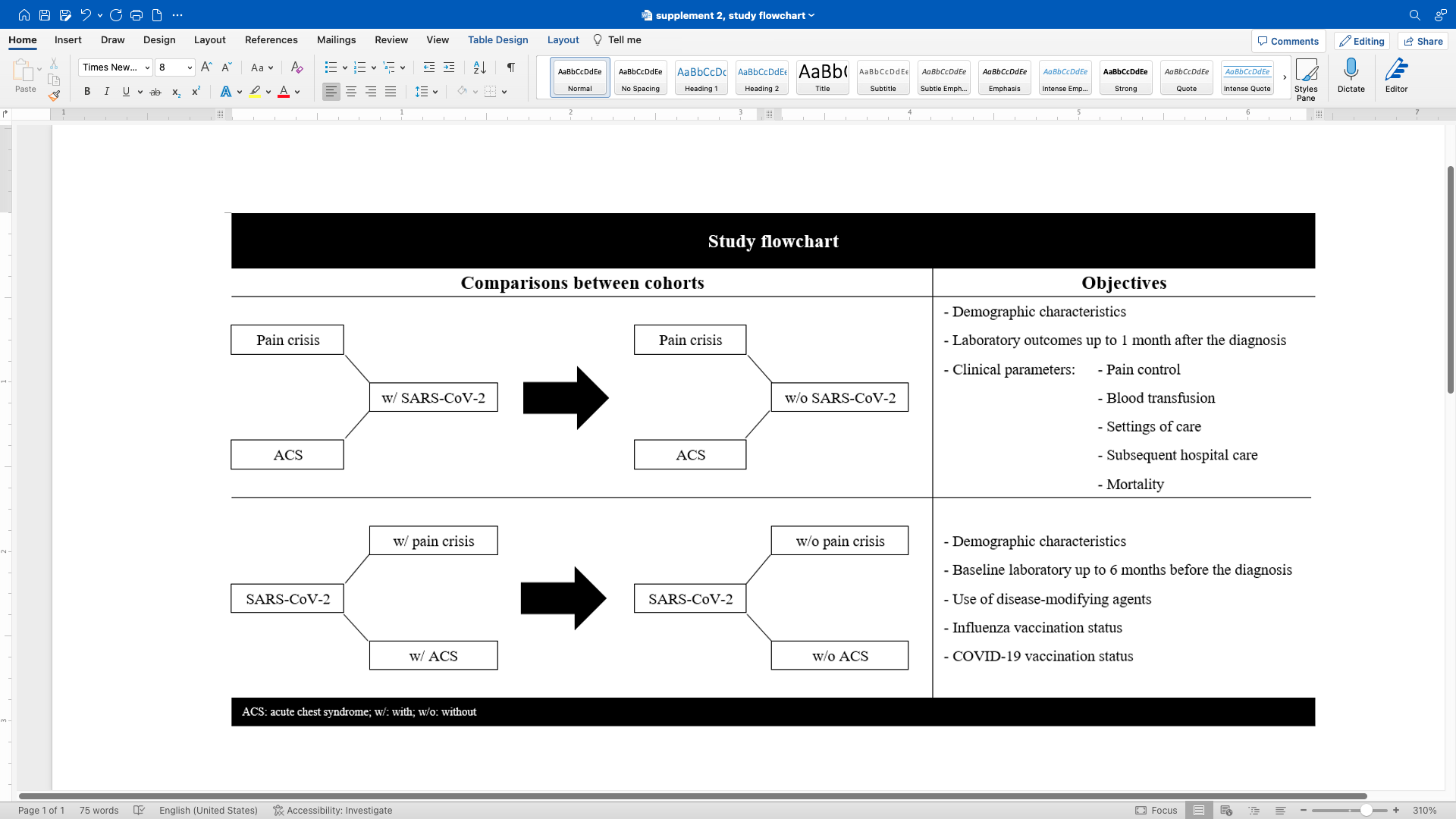 | - Demographic characteristics  - Baseline laboratory up to 6 months before the diagnosis  - Use of disease-modifying agents  - Influenza vaccination status  - Covid-19 vaccination status |
| ACS: Acute chest syndrome; w/: with; w/o without | |
